# Intraocular *Gnathostoma spinigerum*: a case report

**DOI:** 10.1186/1757-1626-2-9370

**Published:** 2009-12-22

**Authors:** Shreekant Tiwari, Nirupama Chayani, Bibhudutta Rautaraya

**Affiliations:** 1 Department of Microbiology, Hi-Tech Medical College and Hospital, Bhubaneswar, Odisha, India; 2 Department of Microbiology, SCB Medical College, Cuttack, Odisha, India; 3 Department of Microbiology, Hi-Tech Medical College and Hospital, Bhubaneswar, Odisha, India

## Abstract

Live intraocular nematode is a rare occurrence that is mostly reported in South East Asian countries. Herewith we report such a case from Nayagarh district of Odisha. A 28 year old female presented with swelling, redness, lacrimation, pain & diminished vision of left eye since 2 1/2 years. Slit lamp examination revealed a worm piercing iris muscle. The worm was removed by paracentesis of anterior chamber and sent to the Department of Microbiology. It was identified to be *Gnathostoma spinigerum *basing on the typical morphology of its cephalic end. The patient responded completely to oral albendazole therapy.

## Introduction

Gnathostomiasis is a rare zoonotic infection of human beings where man is the paratenic host. Human Gnathostomiasis, caused by ingestion of third stage larva (L3) of the nematode (infective form) which may be found in raw or poorly cooked meat (eg. fresh water fish, chicken, snails, frogs etc.) or in contaminated water. It can involve any organ system but the most common manifestation of infection is localized, intermittent, migratory swelling in the skin and subcutaneous tissue. As human is not a suitable host for the parasite, ingestion of infected fish causes migration of the infective form from stomach via intestine to dermal, subcutaneous tissue and often associated with localized pain, pruritus and erythema. Ocular involvement may occur years after initial infection. A total of 12 cases of intraocular gnathostomiasis have been reported in literature until1994 [1]. Till date 6 such cases of intraocular gnathostomiasis have been reported from India. Herewith we report such a case from a lady belonging to slum area of Nayagarh district of Odisha. In this patient a live worm was found to be present in the anterior chamber of the left eye. The case is reported because of its rarity and clinical importance and we believe that this is the first documented case from Odisha.

## Case presentation

A 28 year old female belonging to a slum area of Nayagarh district of Odisha, came to ophthalmology outpatient department with chief complain of swelling, redness, lacrimation, pain and diminished vision of left eye since 2 1/2 years. The symptoms were insidious in onset and progressive in nature. The patient was a house-wife, non-vegetarian, and had no pet animals. On general examination there was no oedema, pallor, organomegally and lymphadenopathy. Examination of the left eye revealed congestion, redness, lacrimation, & swelling but the right eye was normal. Slit lamp examination of the left eye revealed a live worm piercing the iris muscle whose movement was recorded in video. The conjunctival fornices and the cornea were normal. Ocular movement and fundoscopy of both the eye were normal. The worm was removed by paracentesis of anterior chamber and sent in normal saline to the department of Microbiology.

**Figure 1:**
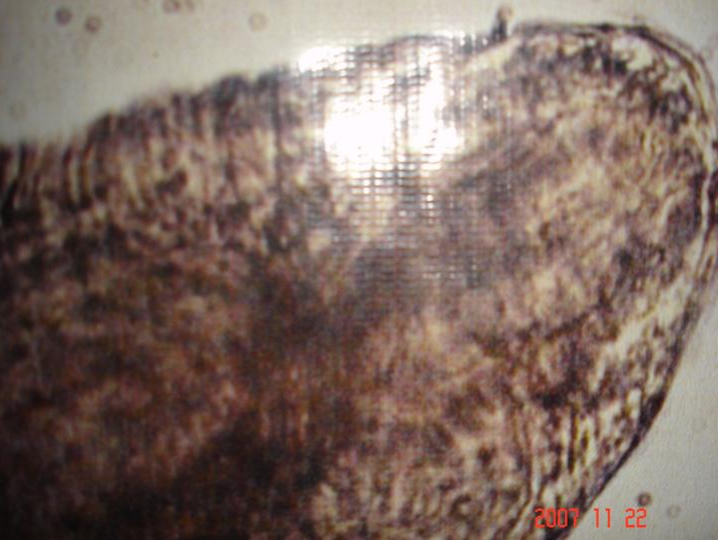
**Tail end of the worm**.

**Figure 2:**
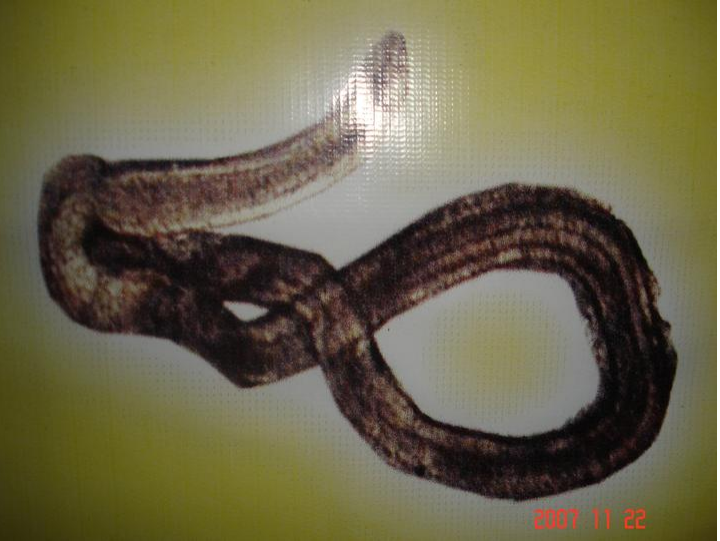
**Gnathostoma spinigerum**.

**Figure 3:**
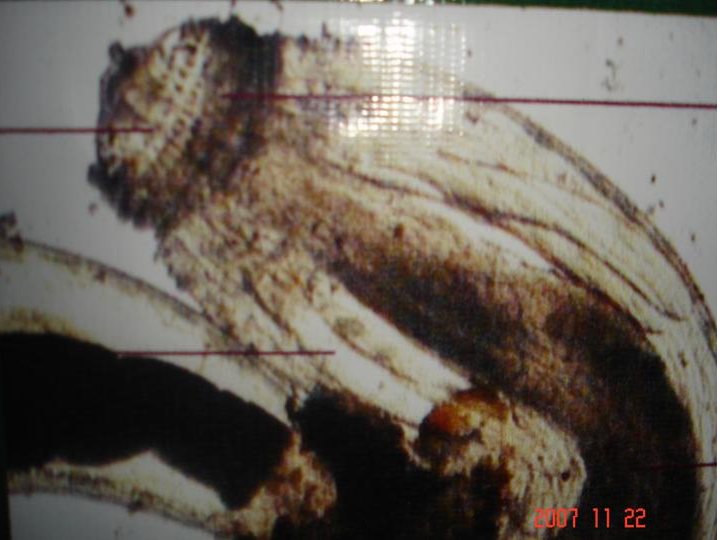
**Cephalic end of the worm**.

Under low & high power magnification morphology of the parasite was studied. The worm was found to be approximately 1.5 to 2.0 cm in length, having a typical head bulb with five circumferential rows of hooklets and fine cuticular spine on body surface [Figure 1, Figure 2 and Figure 3].

Routine examination of the blood revealed normal laboratory parameters except slightly raised eosinophil count (6%). Both thick & thin peripheral blood smear were negative for Microfilaria, Loa-loa, Ascaris, Dirofilaria, Onchocerca etc. No ova, cyst or egg were found in the stool microscopy for three consecutive days. A pair of salivary gland and esophagus was distinctively visible. Basing on the above findings the worm was diagnosed to be a male larva (L3) of *Gnathostoma spinigerum*. The patient was treated with oral albendazole 400 mg daily for 21 days along with tapering dose of oral corticosteroids and responded completely.

## Discussion

Live intraocular nematode is a rare occurrence and mostly reported from Southeast Asian countries, particularly Thailand & Japan [2]. The first human case of intraocular Gnathostomiasis was reported in Thailand [3]. The infection is more common in women than men, perhaps owing to transmission via skin penetration during preparation of food or from contaminated water containing infected copepods [4]. In our case the infection may be acquired by drinking of contaminated water or eating of partially cooked meat or fish as the patient was illiterate and belongs to a slum area. Third stage larvae cannot mature in humans, but they may remain alive up to 10 years [5]. In humans, they may migrate to various organs, including the eye but the mode of entry of *Gnathostoma *larva into the eye is not clearly known.

*Gnathostomes *is one of the causes of larva migrans and may produce fatal eosinophilic myeloencephalitis [6]. If the parasite migrated to CNS it may cause cerebrovascular accident. Infection can be prevented by avoiding the intake of raw or partially cooked meat, fish, snails etc. and contaminated drinking water. Proper sewage disposal and treatment of drinking water may prevent the spread of infection in community. Therapeutic success depends on early & complete surgical removal, which could be life saving, because no anti parasitic drugs are available to treat the ocular involvement [7].

## Abbreviations

CNS: (Central nervous system); L3: (third stage of larva).

## Consent

Written informed consent was obtained from the patient for publication of this case report. A copy of the written consent is available for review by the Editor-in-Chief of the journal.

## Competing interests

The authors declare that they have no competing interests.

## Authors' contributions

ST participated in preparation of the manuscript and review of the patients' medical records. NC conducted the literature review and participated in preparation of the manuscript. BDR participated in preparation of the manuscript. All authors read and approved the final manuscript.

## References

[B1] Biswas J Gopal L Sharma T Badrinath SS Intraocular *Gnathostoma spinigerum*, clinico-pathological study of two cases with review of literature Retina 1994 14 438 444 10.1097/00006982-199414050-00009 7899720

[B2] Nawa Y Historical review and current status of *gnathostomiasis *in Asia Southeast Asian J Trop Med Public Health 1991 22 217 219 1822889

[B3] Rhithibaed C Daengsvang S A case blindness caused by *Gnathostoma spinigerum* J Med Assoc Thai 1937 19 840 845

[B4] Daengsvang S Infectivity of Gnathostoma spinigerum larvae in primates J Parasitol 1971 57 476 8 10.2307/3277896 4996975

[B5] Basak SK Sinha TK Bhattacharya D Hazra TK Parikh S Intravitreal live *Gnathostoma spinigerum* Indian J Ophthalmol 2004 52 57 58 15132381

[B6] Bunang T Comer DS Punyagupta S Eosinophilic myeloencephalitis caused by *Gnathostoma spinigerum*, Neuropathology of nine cases J Neurol Sci 1970 10 419 434 10.1016/0022-510X(70)90023-7 5444893

[B7] Barua P Hazarika NK Barua N Barua CK Choudhury B *Gnathostomiasis *of the anterior chamber Indian J of Medical Microbiol 2007 25 276 278 10.4103/0255-0857.34775 17901651

